# Large Left Shamblin Type III Carotid Body Tumor With Tracheopharyngeal Deviation and Midline Shift: A Case Report

**DOI:** 10.7759/cureus.78935

**Published:** 2025-02-13

**Authors:** Amir Akhavan, John Crawford, Charles A West

**Affiliations:** 1 Vascular Surgery, TCU Burnett School of Medicine, Texas Health Harris Methodist Hospital, Fort Worth, USA

**Keywords:** carotid artery surgery, carotid body tumor, extra-adrenal paraganglioma, head and neck tumors, shamblin type iii, vascular mass excision

## Abstract

A 46-year-old female was referred for treatment of a large left parapharyngeal mass, incidentally discovered and later confirmed as a Shamblin type III carotid body tumor (CBT). There was no family history of CBTs. The patient exhibited no clinical signs of catecholamine excess, and both serum and urine catecholamine levels were normal. CT and CT angiography revealed a 4.2 cm mass arising from the carotid bifurcation, causing significant medial deviation of the trachea and pharynx. Ultrasound imaging showed complete encasement of the carotid artery and bulb by the tumor. Surgical resection was successfully performed via a longitudinal left neck incision, without the need for preoperative embolization, mandibular dislocation, or complex vascular reconstruction of the carotid bifurcation. This case underscores the importance of early detection and timely intervention by an experienced vascular surgical team to manage these complex tumors before they cause significant compression of neck structures, necessitating a more challenging procedure.

## Introduction

Carotid body tumors (CBTs) are rare neuroendocrine neoplasms that originate from the paraganglion cells of the carotid body, which are derived from embryonic neural crest tissue [1,2]. These highly vascular tumors typically arise at the bifurcation of the internal and external carotid arteries. While most CBTs are nonfunctional, some may secrete catecholamines, leading to systemic symptoms such as hypertension, palpitations, sweating, and headaches, although these manifestations are uncommon in head and neck paragangliomas.

Histologically, CBTs display a characteristic “zellballen” growth pattern, consisting of chief cells surrounded by sustentacular cells, which are believed to be modified Schwann cells [1]. Less common histological subtypes include nerve sheath and endothelial-based tumors. Most patients remain asymptomatic, with CBTs often detected incidentally during imaging or physical examination. Familial cases account for approximately 10% of CBTs, and genetic testing for succinate dehydrogenase (SDH) mutations is recommended for at-risk family members [2].

Although the malignancy rate of CBTs is low, diagnosis is sometimes made after metastatic spread, highlighting their potential for aggressive behavior despite their generally benign nature [3]. Surgical resection remains the primary treatment, with the Shamblin classification system guiding surgical complexity based on the tumor’s relationship to the carotid vessels. Shamblin type I tumors are small and easily resectable, whereas Shamblin type III tumors encase the carotid bifurcation and present significant surgical challenges. Postoperative local recurrence is rare.

## Case presentation

A 46-year-old female with hypertension, type 2 diabetes mellitus, and obstructive sleep apnea was undergoing a workup for hyperprolactinemia when a large left neck mass was discovered. CT of the head and neck revealed a sizable parapharyngeal mass at the left carotid bifurcation, causing marked medial deviation of the trachea and pharynx. The patient’s symptoms included headaches, photophobia, and moderate dysphagia. She denied neck pain, difficulty breathing, or palpitations. Serum and urine catecholamine levels were within normal limits, and genetic testing for SDH mutations was not performed.

**Figure 1:**
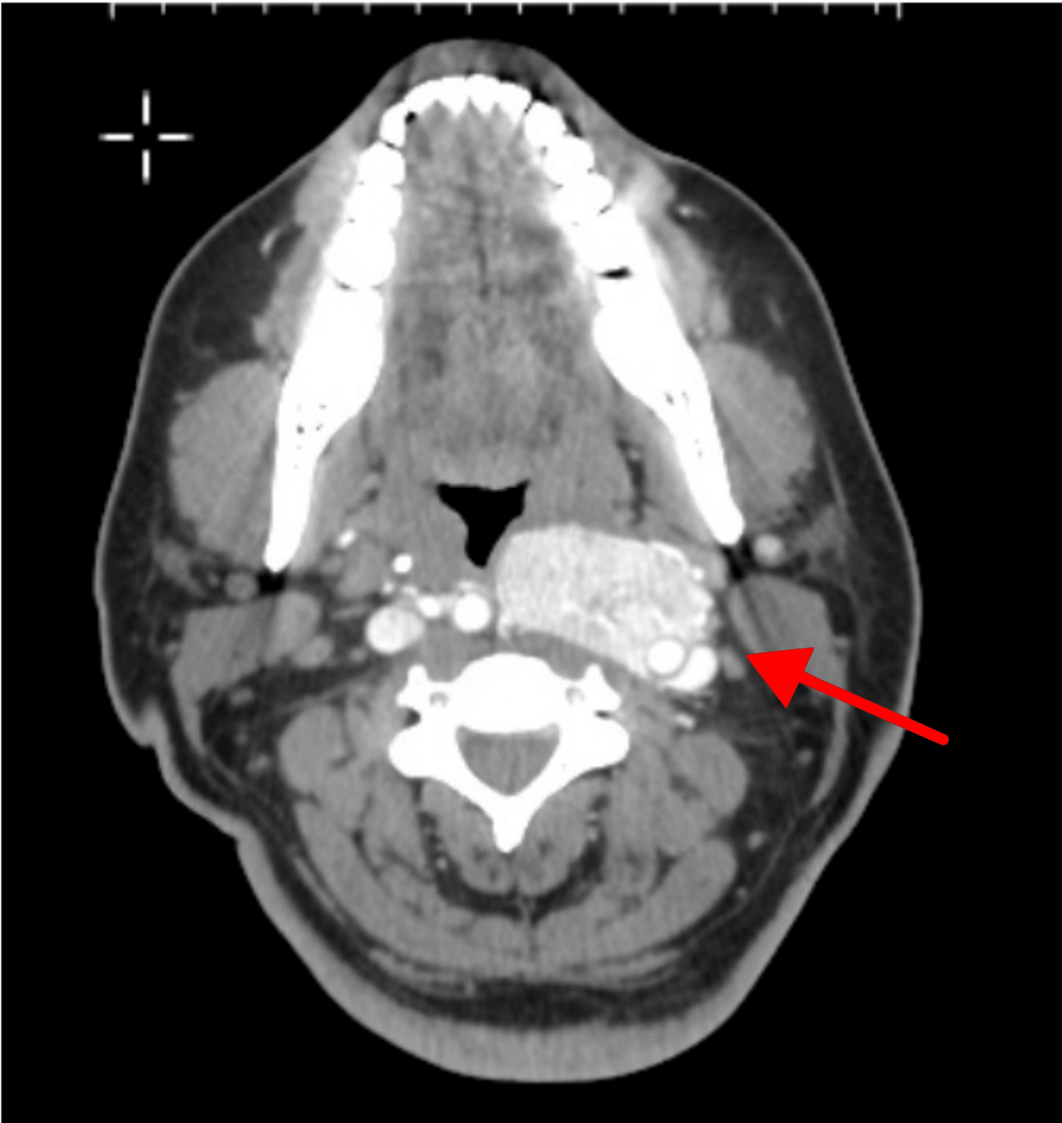
CT angiography showing an axial image of a CBT with tracheopharyngeal compression CBT, carotid body tumor

**Figure 2:**
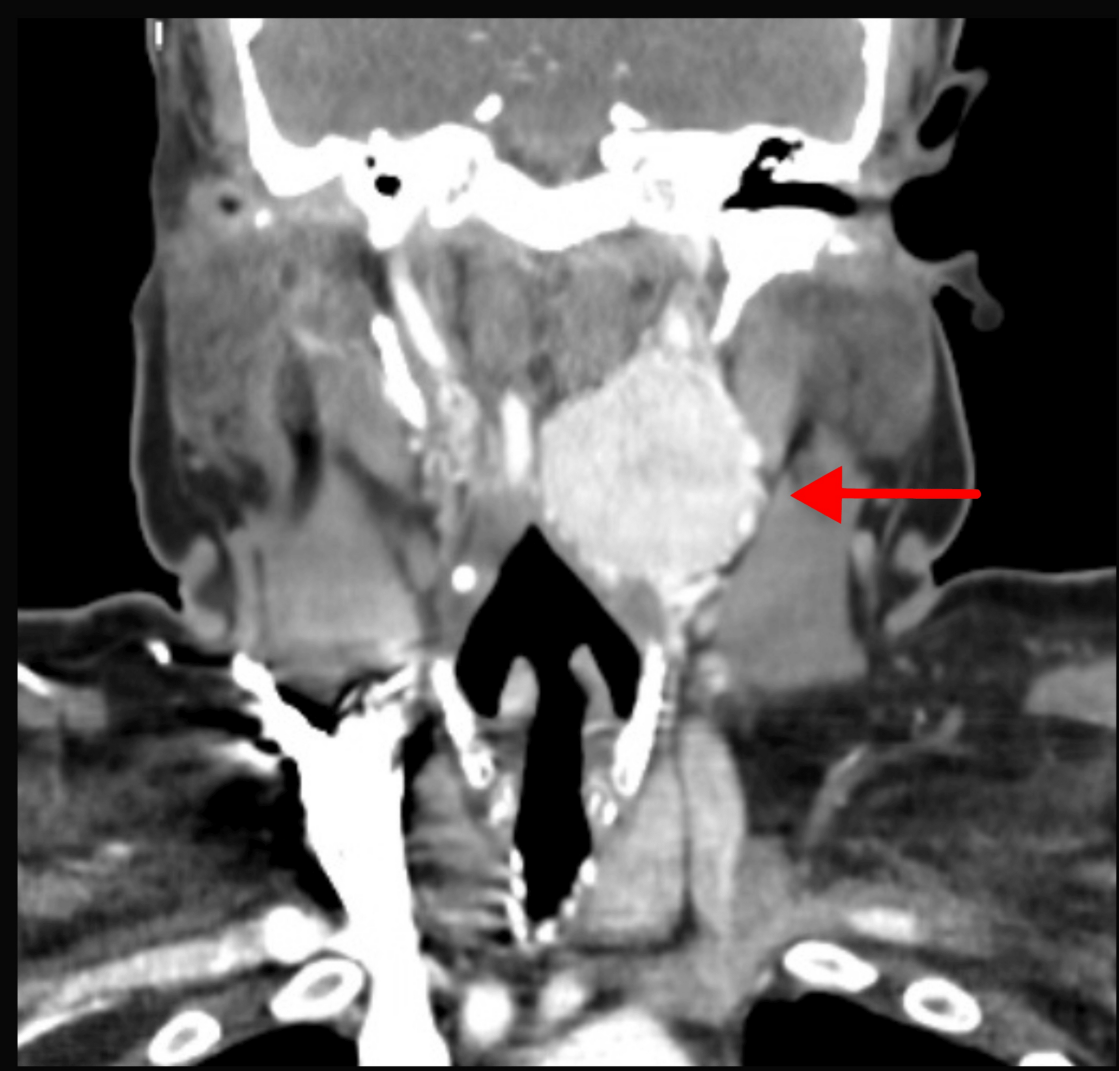
CT angiography showing a coronal image of a Shamblin type III CBT CBT, carotid body tumor

**Figure 3:**
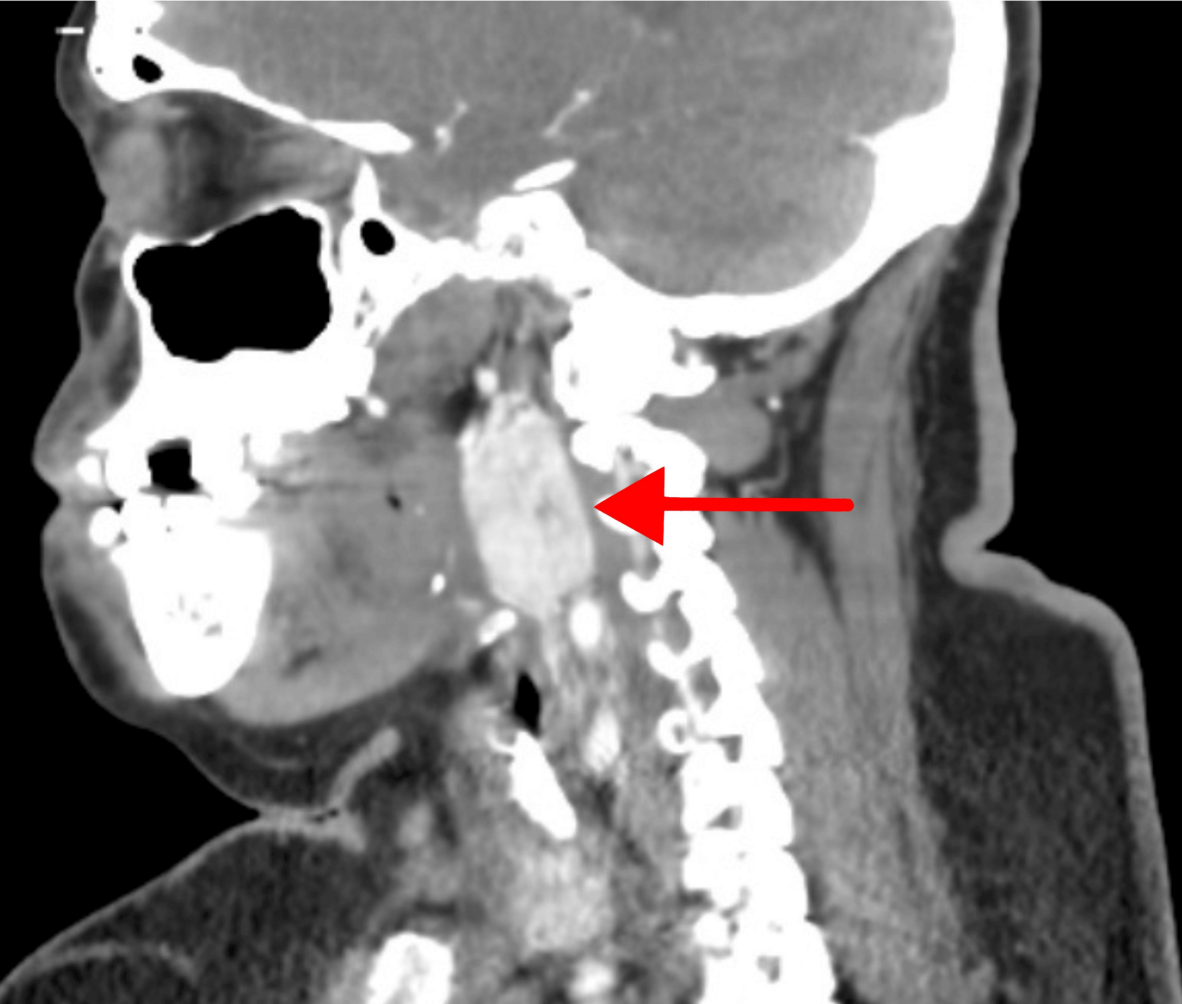
CT angiography showing a sagittal image of a CBT extending to the base of the skull CBT, carotid body tumor

Ultrasound confirmed a vascular mass encircling the left carotid bifurcation. CT angiography (Figure 1, Figure 2, Figure 3) demonstrated a 4.2 × 4.0 cm hypervascular neck mass consistent with a CBT involving the left carotid artery bifurcation. The tumor completely encased the common carotid artery and bulb, and both its size and extent were consistent with a Shamblin type III CBT.

In anticipation of the need for high exposure of the internal carotid artery above the tumor, nasotracheal intubation was performed. The right thigh was also prepped and draped to allow for a potential saphenous vein harvest if vascular reconstruction became necessary.

Surgical resection of the large mass was performed using loupe magnification through a longitudinal left neck incision along the medial border of the sternocleidomastoid muscle. Proximal control of the common carotid artery was established first, followed by distal control of the internal carotid artery above the tumor after dividing the ascending pharyngeal branch of the external carotid artery and the digastric muscle. The left external carotid artery was sacrificed and oversewn, improving medial exposure and effectively devascularizing the tumor, which facilitated resection.

**Figure 4:**
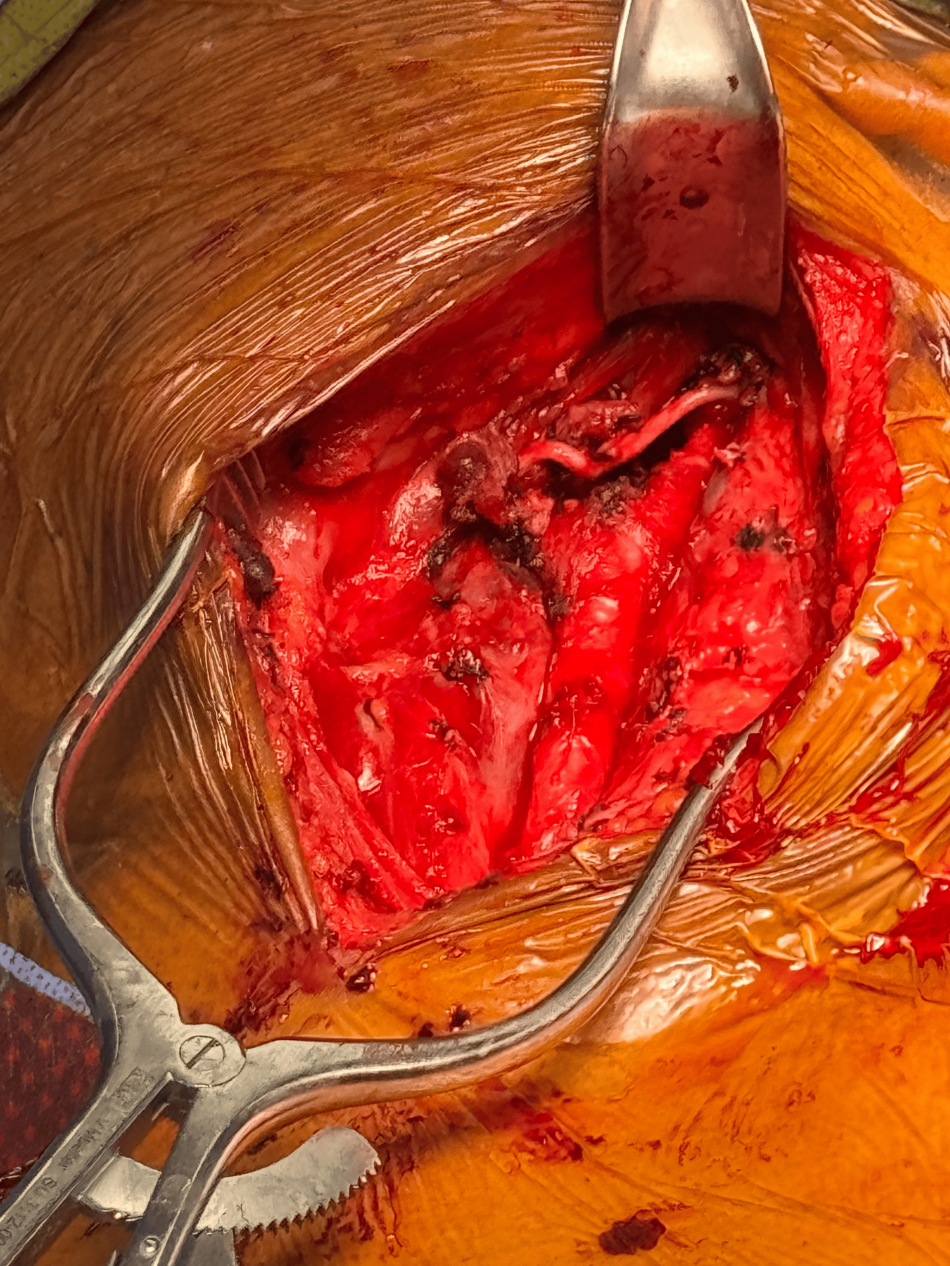
Intraoperative image showing the left common carotid artery and left internal carotid artery, with ligation of the left external carotid artery and exposure of cranial nerves

Bipolar cautery was used to perform a periadventitial dissection of the overlying tumor tissue from the surface of the distal common and proximal internal carotid arteries. Cranial nerves were identified and preserved (Figure 4). A complex carotid reconstruction was not necessary in this case.

The patient’s postoperative course was complicated by transient dysphagia, which required two weeks of nasoenteric feedings. Her full recovery was facilitated by a short course of speech therapy and oral nutritional support.

## Discussion

CBTs account for approximately 0.3% of all paragangliomas, with an incidence of 1-2 per 100,000 individuals. These rare neoplasms originate from chemoreceptor cells at the carotid bifurcation and can grow significantly before clinical detection. Most patients are asymptomatic at presentation, with only 12% reporting neck pain and 1.6% experiencing cranial nerve dysfunction [2]. Ten percent of these tumors are familial, 8.7% can be bilateral, and the reported malignancy rate is 5%, typically diagnosed after metastases are detected [4]. Current guidelines recommend genetic testing for SDH mutations, particularly in familial cases and patients with multiple tumors [2].

Surgical resection remains the primary treatment but is often challenging due to the tumor’s hypervascularity and close relationship with the carotid vessels and cranial nerves. In 1971, Shamblin et al. classified CBTs into three types: Type I tumors are confined to the carotid bifurcation without invading the walls of the internal or external carotid artery. Type II tumors extend below the carotid bifurcation and partially envelop the bifurcation vessels. Type III tumors completely encase the carotid bifurcation and distal common carotid artery, making surgical excision technically demanding [5].

Complications from CBT surgery include cranial nerve injuries, vascular injuries, bleeding, stroke, and, rarely, persistent hypotension [4]. Various adjunct techniques have been described to facilitate the safe removal of large Shamblin type III CBTs, including preoperative tumor embolization, staged procedures, mandibular dislocation, and complex carotid bifurcation reconstruction with autogenous vein grafts, often requiring EEG monitoring and carotid shunting [6]. Approximately 30.2% of CBT resections require revascularization, with the rate increasing for Shamblin type II and type III tumors compared to Shamblin type I. Local recurrence is rare when dissection is performed in the periadventitial plane.

Bipolar tissue-sealing devices, such as LigaSure, can aid in safely dissecting these tumors from the carotid vessels after dividing the external carotid artery. Although external carotid artery division is not always necessary, it is typically required when improved exposure or devascularization is critical. These devices also help reduce operative time and blood loss.

This case highlights several important aspects of CBT management. Firstly, it demonstrates that these tumors can reach substantial sizes before detection, reinforcing the importance of early diagnosis and intervention. A unique feature of this case is the significant medial deviation and tracheopharyngeal compression, along with carotid encasement, emphasizing the potential for mass effects on surrounding structures. Another key takeaway is the successful surgical management of a complex tumor with preservation of critical neurovascular structures, providing a valuable reference for clinicians handling similar cases. This case also illustrates that even large Shamblin type III tumors can be successfully resected with careful planning and meticulous surgical technique by an experienced vascular surgery team, without the need for preoperative embolization or mandibular dislocation.

Overall, this case report underscores the importance of a comprehensive approach to CBTs, from early detection using advanced imaging to skilled surgical management and attentive postoperative care. It serves as a reminder of the potential for these rare tumors to remain clinically silent until they reach considerable sizes and highlights the need for timely intervention to prevent complications and ensure optimal patient outcomes.

## Conclusions

This case demonstrates that CBTs can grow significantly and compress critical neck structures before clinical detection. Proper imaging studies are essential for surgical planning and identifying asymptomatic contralateral tumors. Even a large CBT completely encasing the carotid bifurcation can be safely resected in a single operation by an experienced vascular surgical team, without the need for staged procedures, preoperative embolization, mandibular dislocation, carotid bifurcation resection, or complex vascular reconstruction.
